# Demographic and clinical risk factors associated with severity of lab-confirmed human leptospirosis in Colombia, 2015–2020

**DOI:** 10.1371/journal.pntd.0011454

**Published:** 2023-07-05

**Authors:** Eliana L. Parra Barrera, Solmara Bello Piruccini, Karina Rodríguez, Carolina Duarte, Marisa Torres, Eduardo A. Undurraga

**Affiliations:** 1 Facultad de Medicina, Pontificia Universidad Católica de Chile, Santiago, Región Metropolitana, Chile; 2 Multidisciplinary Initiative for Collaborative Research on Bacterial Pathogens and Resistance (MICROB-R), Santiago, RM Chile; 3 Grupo de Virología, Instituto Nacional de Salud, Bogotá, Colombia; 4 Grupo de Microbiología, Instituto Nacional de Salud, Bogotá, Colombia; 5 Escuela de Gobierno, Pontificia Universidad Católica de Chile, Santiago, RM, Chile; 6 CIFAR Azrieli Global Scholars program, CIFAR, Toronto, Canada; 7 Research Center for Integrated Disaster Risk Management (CIGIDEN), Santiago, RM Chile; DotLab, UNITED STATES

## Abstract

**Background:**

Leptospirosis is a common zoonoses and is a major global public health threat. Most cases are mild, typically presenting as a non-specific acute febrile illness. However, leptospirosis can have life-threatening manifestations, including pulmonary hemorrhage syndrome, and acute kidney injury. In Colombia, notification and lab-confirmation of suspected human cases are mandatory. However, little is known about the demographic and clinical factors associated with severe leptospirosis, which could help to reduce clinical complications and mortality. Our aim was to identify risk factors associated with severe leptospirosis, intensive care unit (ICU) admission, and mortality in lab-confirmed cases in Colombia, 2015–2020.

**Methods and findings:**

We analyzed 201 lab-confirmed human leptospirosis cases by microagglutination test. We used a logistic regression to identify the demographic and clinical risk factors associated with severe leptospirosis, admission to ICU, and death. Most leptospirosis confirmed cases occurred in men (85.6%); the mean age was 36.7 years. We classified severe cases (43.3%) by clinical manifestations as renal (29.9%) and liver (27.4%) failure, multiple-organ failure (24.4%), septic shock (24.4%), Weil syndrome (18.4%), pulmonary hemorrhage (18.4%), and meningitis (2.5%), admitted to the ICU (30.3%), and fatal (8.5%). Clinical conditions associated with severe leptospirosis were dyspnea (OR: 5.54; 95% CI: 1.46 to 20.98), tachycardia (OR:9.69; 95% CI: 15.96 to 58.8), and rash (OR: 10.25; 95% CI: 25.01 to 42.08).

**Conclusions:**

We identified demographic characteristics and clinical symptoms associated with severe leptospirosis in Colombia. We hope these results can support clinicians in providing timely treatment to leptospirosis patients to avoid preventable medical complications or deaths.

## Author summary

Leptospirosis is a neglected tropical disease and a public health concern worldwide. Infection in humans usually occurs through direct or indirect contact with the urine of infected animals. Most leptospirosis cases are mild, but between 5–15% of cases can be severe and even fatal, primarily due to delays in diagnosis, misrecognition, inadequate treatment, or pathogenicity of some strains. The factors associated with severity could be used to support triaging and avoid preventable medical complications or death. Colombia is an endemic country for leptospirosis, with mandatory laboratory surveillance. Our objective was to determine the demographic and clinical risk factors associated with severe leptospirosis. We used a unique dataset of 201 lab-confirmed leptospirosis cases notified to the National Reference Laboratory with a reported clinical history from several regions in Colombia. The cases presented severe illness (43.3%), ICU admission (30.3%), and fatal (8.5%). Severe leptospirosis was associated with dyspnea, tachycardia, and rash; fatal cases were associated with jaundice. We hope these results raise awareness among healthcare workers and support clinicians in avoiding preventable medical complications or death in human leptospirosis in Colombia and elsewhere.

## Introduction

Leptospirosis is a bacterial zoonosis with a worldwide distribution [1,2], with an estimated one million human cases and 60,000 deaths annually [1]. Infection in humans usually occurs through direct or indirect contact with the urine of infected animals [3]. Pathogenic leptospires penetrate the skin through abrasions and can rapidly migrate from the dermis, spreading through the bloodstream, to remain in different organs [3–5]. Human leptospirosis can present from subclinical infection to a severe syndrome with multiple organ failures requiring ICU and high mortality [6–8]. The disease is, in most cases, asymptomatic or mild, with a wide variety of clinical manifestations and flu-like symptoms (sudden onset of fever, headache, muscle pain or myalgia, and gastrointestinal involvement). However, between 5% to 15% of infections can result in severe clinical manifestations [3], including multiple organ damage (mainly kidneys and liver), severe pulmonary syndrome [5,7,9], meningitis, or Weil’s syndrome (fever with bleeding tendencies, liver dysfunction, and acute kidney failure). Mortality can be significant due to delays in diagnosis, misrecognition, inadequate treatment, or pathogenicity of some strains [10,11]. The case fatality rate for patients with severe leptospirosis is approximately 5% to 15% [12]. However, it can be greater than 50% for patients with pulmonary hemorrhagic syndrome, which occurs in 20–70% of patients with severe illness [6].

Various clinical signs and symptoms may occur in leptospirosis patients [1,13]. At the outset, non-specific presentation makes the clinical diagnosis difficult. The World Health Organization (WHO) suggests that symptoms consistent with leptospirosis include fever, severe headache, muscle pains, conjunctival injection, jaundice, general discomfort, stiff neck, shaking chills, abdominal pain, anorexia, nausea, vomiting, diarrhea, oliguria/anuria, hemorrhages, skin rash, photophobia, cough, cardiac arrhythmia, hypotension, mental confusion, psychosis, delirium [14]. Also, unusual clinical conditions have been reported in leptospirosis, mainly involving the lungs [15], cardiovascular, gastrointestinal, neural, ocular, and other systems [16]. These include myelitis, rhabdomyolysis, hematologic parameters (normochromic normocytic anemia, reticulocytopenia, thrombocytopenia, hypogonadism) [17], behavior change, glomerulonephritis, bilateral paralysis, sinus thrombosis, pancreatitis with necrosis, brainstem involvement, and paraparesis [18].

Several prognostic clinical factors could be used to predict risk development of severe disease in human leptospirosis [19–24]. In tropical areas, due to the presence of infectious diseases very similar to leptospirosis, physicians need to be aware of prognostic factors associated with severe leptospirosis. Worldwide, the main factors associated with clinical complications or severity include the serovar of the infecting pathogenic *Leptospira* spp. species, host susceptibility, environment, and host behavior [21,25–27]. Among the independent prognostic factors for severe or fatal leptospirosis have been described age, oliguria, arrhythmia, hemorrhage, jaundice, renal failure, hyperkalemia, acute respiratory distress syndrome, pulmonary hemorrhage, pulmonary rales, elevated bilirubin, hypotension, and altered mental status [20,28–35].

In Colombia, leptospirosis is considered an endemic disease. Suspected human cases must be notified to health authorities, and laboratory confirmation at the National Reference Laboratory has been mandatory since 2007 [36]. However, leptospirosis is substantially underreported or misdiagnosed due to limited access to adequate diagnostic tests, lack of clinical awareness, and because most cases of leptospirosis are mild and typically present as an undifferentiated fever [1,11,37–39]. Delays in diagnosis, misrecognition, lack of clinical awareness, limited access to healthcare, or clinical referral to secondary or tertiary healthcare centers over great distances, may result in preventable clinical complications and sometimes death. Perez-García et al. reported that 29.7% of hospitalized pediatric patients developed severe leptospirosis [40]. Studies in tertiary hospitals have reported that 16% to 34% of leptospirosis patients required admission to an ICU [13,41] or were fatal [40,42]. However, factors associated with severe leptospirosis are still unknown or not well-established. Most leptospirosis research focuses on specific prevalence studies. Few studies have examined hospital and ICU admissions and the clinical evolution of patients. This is important, because understanding risk factors may support a more timely and adequate patient triaging and treatment, reducing avoidable clinical complications and mortality.

Our objective was to identify the clinical and demographic factors associated with severe leptospirosis in laboratory-confirmed human leptospirosis cases in Colombia. We used a unique dataset of lab-confirmed cases in Colombia between January 2015 and December 2020. Clinicians in Colombia, particularly in regions with limited access to health systems or resources, face substantial challenges in understanding the factors that indicate potentially severe leptospirosis cases who may need to be transferred to tertiary care centers or intensive care units. We hope our results help inform their clinical decisions.

## Methods

### Ethics statement

The study used anonymized secondary epidemiological laboratory surveillance data with authorization from the Microbiology Group at the National Institute of Health, Colombia. The Scientific Ethics Committee for Health Sciences at the Pontificia Universidad Católica de Chile reviewed and approved study protocol (Resolution Act: protocol ID 210418003 of August 31, 2021). Analyses were done following protected health information guidelines and regulations in Colombia and Chile. Personal information (name, address, telephone, among other data that could be personally identifiable) was not used in the study. The study did not require informed consent.

### Study location and population

We conducted a cross-sectional retrospective study design. We used data from lab-confirmed human leptospirosis reported to the National Reference Laboratory, Microbiology Group, at the National Institute of Health. The surveillance laboratory network consists of 33 public health laboratories in each department. Medical centers must notify all suspected leptospirosis cases through the National Surveillance System (SIVIGILA; Sistema de Vigilancia en Salud Pública).

The epidemiological surveillance protocol for leptospirosis defines a suspected case as having fever (>38°C) in the previous three weeks and at least two signs or symptoms, including headache, myalgia, conjunctivitis, arthralgia, vomiting, diarrhea, back pain, chills, intraocular pain or photophobia, and skin rash. The patient’s clinical and epidemiological history is also considered, including potential environmental exposures (floods, mud, wells, streams, lakes, or rivers), work with animals, and outdoor or recreational exposures [43]. If a leptospirosis case is suspected, local medical centers perform an enzyme-linked immunosorbent assay or ELISA, with the detection of immunoglobulins M (IgM) from two serum samples (in the acute and convalescent phases of the disease, between 10 to 15 days). The surveillance protocol establishes that only cases with reactive sera for detecting ELISA-IgM antibodies should be reported to public health laboratories. These laboratories send the sera samples and the corresponding epidemiological report to the Reference Laboratory for laboratory confirmation [43].

Leptospirosis case confirmation was done using a microscopic agglutination test (MAT) to detect IgM and IgG antibodies with a panel of 29 serovars of pathogenic species and a control serovar (*Leptospira biflexa* serovar Patoc, Patoc I strain). A leptospirosis case is considered positive if it presents agglutination in the MAT for at least one serovar of the panel with 1) a titer of 400 or more in individual or paired samples or four-fold increase in MAT titer between acute and convalescent serum samples.

### Data collection and management

Demographic and epidemiological data were collected based on the Ministry of Health’s form 455 for leptospirosis surveillance and the clinical report. Form 455 reports demographic data, including sex, age, area of occurrence, dates of onset of symptoms, and hospitalizations; signs, including fever, headache, hepatomegaly, jaundice, myalgias; and epidemiological risk factors associated with leptospirosis infection (S1 File). Clinical diagnoses were assessed based on case report forms (i.e., leptospirosis form and clinical history).

Human leptospirosis cases were classified for the analysis of outcome variables as severe disease, UCI admission, and non-survivors cases. An accepted standard criterion has not been established to define severe leptospirosis, which is usually associated with the dysfunction of multiple organs. Therefore, we defined severe disease based on the case’s clinical report if at least one of the following clinical conditions were reported during the hospital stay: septic shock, Weill syndrome, kidney failure, liver failure, pulmonary hemorrhage, meningitis, multi-organ failure, and admission to the ICU. Mortality was assumed when a fatal case was reported and/or if fresh or paraffin tissue samples were remitted to the laboratory.

### Statistical analysis

We first show descriptive statistics of demographic, epidemiological, and clinical variables. We then estimated the bivariate association of demographic, epidemiological, clinical variables, and our outcomes of interest (severe leptospirosis) using a Chi-Square (χ^2^) or Fisher’s exact test. Next, we used logistic regressions to determine the association between our outcomes of severe leptospirosis with the explanatory variables of risk. We selected explanatory variables based on biological plausibility, clinical relevance, and statistical significance in the univariate analysis, including variables with p<0.20 in our final model. We examined multicollinearity in our model using variance inflation factor (VIF) (<4.0) and tolerance (>0.2). Finally, we estimated odds ratios (OR) and 95% confidence intervals (CI). Multiple receivers operating characteristic (ROC) curves were constructed. The Ministry of Health supplied anonymized data with no sensitive patient information in an Excel spreadsheet (Microsoft Corporation). The analysis was done using IBM SPSS v.26 and R with the open-source interface Jamovi Solid v.2.2.5 (https://www.jamovi.org).

## Results

A total of 3,535 suspected human leptospirosis cases were notified from 22 public health laboratories in Colombia. Among these cases, 880 (25%) were leptospirosis lab-confirmed cases by MAT. We retrospectively identified all laboratory-confirmed cases with clinical data available. Of these, 679 (77.2%) had no clinical report. Our analysis was based on 201 lab-confirmed leptospirosis cases with a clinical report. Therefore, our analysis was based on 201 lab-confirmed leptospirosis cases with a clinical report. Public health laboratories collected serum samples from the medical institutions in each region. Most reports were sent from the health public laboratories in Tolima (33.8%), Santander (12.9%), Risaralda (9.0%), Cundinamarca (8.5%), Bogotá (7.0%), and Huila (6.0%).

For lab-confirmation, two serum samples (from acute and convalescent phases of the disease) were used in 185 cases (92%). IgM ELISA test, performed at the primary health institutions, was reported for 152 (75.6%) cases, with 140 (69.7%) reactive results for IgM antibodies. The MAT-confirmed cases included samples with 4-fold increases in titer (92%), and titers greater than 400 (8%). Serological analysis of all MAT-positive samples showed reactivity to twenty-seven serovars. The most frequent were Australis (22.4%), Ballum (10.4%), Autumnalis (9.0%), Wolfii (6.5%), Tarasovi (6.0%), Icterohaemorrhagiae (5.5%), Pyrogenes (4.5%), Bataviae (3.5%), Hebdomadis (3.0%), and others (29.4%) (S1 Table). Australis was the most frequently observed in severe leptospirosis (20.7%), UCI admission (21.3), and death (23.5%). However, no significant differences were observed in the serovar analysis associated with severe disease, ICU or admission mortality.

Most leptospirosis cases occurred in men (85.6%). The mean age was 36.7 SD± 18.4 years (range 3–89 years). Most cases were residing in urban areas (64.6%) (Table 1). Most cases were unrelated to occupation usually considered at a higher risk for leptospirosis infections (71.3%). Confirmed cases from at-risk occupations included farmers (15.9%), military (8.0%), construction (2.0%), fishermen (0.5%), and veterinarians (0.5%). 30.0% of cases were unemployed and 12.2% were students. A chi-squared test showed no significant association between patients’ demographic characteristics and the severity of illness, ICU admissions, or death. Reported cases with a medical history had a low proportion (8.0%), including, hypertension (3.0%), alcoholism (1.0%), diabetes (1.0%), smoking (1.0%), neoplasia (1.0%), and human immunodeficiency virus infection (1.0%).

**Table 1:** Demographic characteristics of human leptospirosis cases with diagnostic laboratory confirmation (MAT) in Colombia, 2015–2020 (n = 201).

Variables		Total	Severe Leptospirosis	Non-severe Leptospirosis	Non-Survivors	Survivors
		n (%)	n (%)	n (%)	n (%)	n (%)
**Total n (%)**		201	87 (43.3)	114 (56.7)	17 (8.5)	184 (91.5)
Age group	0–9	2 (1.0)	2 (2.2)	0 (0.0)	1 (5.8)	1 (0.5)
	10–19	8 (4.0)	3 (3.4)	5 (4.3)	0 (0.0)	8 (4.3)
	20–29	31 (15.4)	12 (13.7)	19 (16.7)	3 (17.6)	28 (15.2)
	30–39	41 (20.4)	17 (19.5)	24 (21.1)	2 (11.8)	39 (21.2)
	40–49	41 (20.4)	18 (20.6)	23 (20.2)	1 (5.9)	40 (21.7)
	50–59	21 (10.4)	9 (10.3)	12 (10.5)	3 (17.6)	18 (9.8)
	60–69	34 (16.9)	15 (17.2)	19 (16.7)	2 (11.8)	32 (17.4)
	70–79	16 (8.0)	8 (9.2)	8 (7.0)	3 (17.6)	13 (7.1)
	>80	7 (3.5)	3 (3.4)	4 (3.5)	2 (11.8)	5 (2.7)
Sex	Men	172 (85.6)	72 (82.8)	100 (87.7)	14 (82.4)	158 (85.9)
	Women	29 (14.4)	15 (17.2)	14 (12.3)	3 (17.6)	26 (14.1)
Occupational contact with risk	Yes	54 (26.9)	21 (24.1)	33 (28.9)	2 (11.8)	52 (28.3)
	No	147 (73.1)	66 (75.9)	81 (71.1)	15 (88.2)	132 (71.7)
Healthcare system	Contributory^a^	99 (49.3)	42 (48.3)	57 (50.0)	6 (35.3)	93 (50.5)
	Other	102 (50.7)	45 (51.7)	57 (50.0)	11 (64.7)	91 (49.5)
Area	Urban	130 (64.7)	56 (64.4)	74 (64.9)	11 (64.7)	119 (64.7)
	Rural	71 (35.3)	31 (35.6)	40 (35.1)	6 (35.3)	65 (35.3)
Animals at home	Yes	128 (63.7)	50 (57.5)	78 (68.4)	12 (70.6)	116 (63.0)
	No	73 (36.3)	37 (42.5)	36 (31.6)	5 (29.4)	68 (37.0)
Rats in the environment	Yes	93 (46.3)	40 (46.0)	53 (46.5)	7 (41.2)	86 (46.7)
	No	108 (53.7)	47 (54.0)	61 (53.5)	10 (58.8)	98 (53.3)

^a^ The contributory regime links individuals and families to the General Social Security Health System through a previous economic contribution financed directly by the member.

Most cases were hospitalized (98.5%). The time between the onset of symptoms and professional health consultation was 5.4 days (SD = 4.8), with no statistically significant differences between men and women (p = 0.994). Most prevalent symptoms were fever (99.5%), myalgia (87.1%), headache (83.1%), arthralgia (67.7%), abdominal pain (60.2%), jaundice (58.2%), and thrombocytopenia (50.7%) (Table 2).

**Table 2:** Clinical leptospirosis characteristics of lab-confirmation cases in Colombia, 2015–2020 (n = 201).

Characteristic	Total n (%)	Severe Leptospirosis	Non-severe Leptospirosis
Total n (%)	201	87 (43.3)	114 (56.7)
**Initial general symptoms**			
Fever (>38°C)	200 (99.5)	87 (100)	113 (99.1)
Myalgias	175 (87.0)	75 (86.2)	100 (87.7)
Headache	167 (83.0)	68 (78.2)	99 (86.8)
Arthralgias	136 (67.6)	53 (60.9)	83 (73.8)
Chills	36 (17.9)	12 (13.8)	24 (21.1)
**Gastrointestinal**			
Nausea	93 (46.3)	45 (51.7)	48 (42.1)
Abdominal pain	121 (60.2)	48 (55.2)	73 (64.0)
Vomit	83 (41.3)	40 (46.0)	43 (37.7)
Diarrhea	91 (45.3)	48 (55.2)	43 (37.7)
Jaundice	117 (58.2)	55 (63.2)	62 (54.4)
Hepatomegaly	52 (25.9)	26 (29.9)	26 (22.8)
Splenomegaly	63 (31.3)	49 (56.3)	14 (12.3)
Pancreatitis	35 (14.4)	33 (37.9)	2 (1.8)
**Respiratory**			
Cough	44 (21.9)	37 (42.5)	7 (6.1)
Pneumonia	39 (19.4)	38 (43.7)	1 (0.9)
Dyspnoea	65 (32.3)	47 (54)	18 (15.7)
Ventilation	34 (16.9)	33 (37.9)	1 (0.8)
Hematuria	46 (22.9)	42 (48.3)	4 (3.5)
**Renal**			
Oliguria	40 (19.9)	39 (44.8)	1 (0.9)
Dysuria	44 (21.9)	44 (50.6)	0 (0.0)
Proteinuria	38 (18.9)	37 (42.5)	1 (0.9)
Dialysis	37 (18.4)	37 (42.5)	0 (0.0)
Cardiovascular			
Arrhythmia	31 (15.4)	31 (35.6)	0 (0.0)
**Included in the classification of serious illness**			
Tachycardia	50 (24.9)	47 (54.0)	3 (2.6)
Septic shock	49 (24.4)	49 (56.3)	0 (0.0)
Multi-organ failure	49 (24.4)	49 (56.3)	0 (0.0)
Renal failure	60 (29.9)	60 (69.0)	0 (0.0)
Liver failure	55 (27.4)	55 (63.2)	0 (0.0)
Weill síndrome	37 (18.4)	37 (42.5)	0 (0.0)
Pulmonary hemorrhage	37 (18.4)	37 (42.5)	0 (0.0)
Meningitis	5 (2.5)	5 (5.7)	0 (0.0)
**Other**			
Thrombocytopenia	102 (50.7)	58 (66.7)	44 (38.6)
Rash	45 (22.4)	39 (44.8)	6 (5.3)
Hemorrhage	50 (24.9)	47 (54.0)	3 (2.6)
Retro ocular pain	16 (8.0)	15 (17.2)	1 (0.9)
Blood transfusión	39 (19.4)	38 (43.7)	1 (0.9)
Use of vasopressors	51 (25.4)	51 (58.6)	0 (0.0)
Antibiotic administration	134 (66.7)	66 (75.9)	68 (59.6)
Prior consultation	25 (12.4)	11 (12.6)	14 (12.3)
Medical history	13 (8.0)	8 (9.2)	8 (7.0)

Eighty-seven (43.3%) cases met our definition for severe leptospirosis. The average age was 38.2 SD 19.3 years, and most severe cases occurred in men (82.2%). Among the severe cases, 49 (24.4%) reported septic shock. Organ failure included kidney and liver in 60 (29.9%) and 55 (27.4%) cases, respectively. Multiple organ failure was reported in 49 (24.4%) cases. Both, Weil syndrome and pulmonary hemorrhage were described in 37 (18.4%) clinical reports. In five (2.5%) cases, meningitis was suspected. Among all cases reported, 61 (30.3%) were admitted to the ICU. Patients in the ICU admission group have a mean of 38.4 (SD 19.3) years. Age was significantly associated with the ICU admission among leptospirosis patients (OR: 2.97; 95% CI: 1.50–5.88).

The univariate analysis suggests the association of severe leptospirosis with mid sings as, diarrhea (OR:2.04 95% IC: 1.15 to 3.60), cough (OR:10.67; 95% IC: 4.45 to 25.56), retro ocular pain (OR: 22.50; 95% IC: 2.91 to 173.99) and severe complications as thrombocytopenia (platelet count abnormalities) (OR:3.15; 95% IC: 1.76 to 5.64), splenomegaly (OR:8.57 95% IC: 4.26 to 17.24), pancreatitis (OR:32.4 95% IC: 7.5 to 139.9), dyspnea (OR:16.5; 95% IC: 7.58 to 35.90), among others (Table 3).

**Table 3:** Univariate and multivariate analysis of the factors for severe leptospirosis, admission to the intensive care unit ICU, and fatal cases.

Characteristic	Severe Leptospirosis			
	Univariate analysis		Multivariate analysis	
	OR (95% CI)	*p* ^*b*^	OR (95% CI)	*p* ^*b*^
Age	0.99 (0.97–1.0)	0.315	1.00 (0.97–1.03)	0.809
Sex	0.7 (0.32–1.55)	0.384	0.754 (0.18–3.03)	0.691
Myalgias	0.76 (0.33–1.75)	0.530	0.39 (0.10–1.42)	0.155
Headache	0.49 (0.23–1.04)	0.064	0.313 (0.08–1.14)	0.078
Arthralgias	0.56 (0.31–1.02)	0.060	0.929 (0.29–2.9)	0.900
Chills	0.57 (0.26–1.22)	0.148	0.620 (0.17–2.52)	0.468
**Gastrointestinal**				
Nausea	1.36 (0.78–2.39)	0.277	0.635 (13.94–2.89)	0.557
Abdominal pain	0.62 (0.35–1.11)	0.107	0.285 (0.08–0.98)	0.048
Vomit	1.31 (0.74–2.31)	0.349	0.423 (0.07–2.24)	0.312
Diarrhea	**2.04 (1.15–3.60)**	**0.014**	4.44 (0.97–20.17)	0.053
Jaundice	1.54 (0.87–2.73)	0.136	1.818 (0.68–4.85)	0.233
Hepatomegaly	1.36 (0.72–2.57)	0.336	1.95 (0.68–5.63)	0.212
Splenomegaly	**8.57 (4.26–17.24)**	**<0.001**	1.41 (0.33–5.97)	0.638
Pancreatitis	**32.4 (7.5–139.9)**	**<0.001**	4.12 (0.34–48.72)	0.261
**Respiratory**				
Cough	**10.67 (4.45–25.56)**	**<0.001**	0.45 (0.07–2.75)	0.393
Dyspnoea	**16.5 (7.58–35.90)**	**<0.001**	**5.54 (1.46–20.98)**	**0.012**
**Renal**				
Hematuria	**24.12 (8.19–71.11)**	**<0.001**	1.11 (0.07–16.94)	0.938
Oliguria	**86.6 (11.6–647.97)**	**<0.001**	4.25 (0.17–106.28)	0.378
**Others**				
Tachycardia	**40.65 (12.0–137.74)**	**<0.001**	**9.69 (15.96–58.88)**	**0.014**
Thrombocytopenia	**3.15 (1.76–5.64)**	**<0.001**	1.80 (0.68–4.75)	0.233
Retro ocular pain	**22.50 (2.91–173.99)**	**0.002**	4.48 (0.28–71.26)	0.288
Rash	**13.78 (5.47–34.67)**	**<0.001**	**10.25 (25.01–42.08)**	**0.001**
Non-antibiotic Administration	**2.04 (1.109–3.77)**	**0.022**	1.08 (0.41–2.87)	0.868

Antibiotic therapy was not administered to all the cases (66.7%). The cases with antibiotic administration included ceftriaxone (50.0%), doxycycline (14.9%), ampicillin (7.4%), penicillin (5.2%), and others (22.3%). Some cases received two (26.1%) and three (7.4%) antibiotics in the same hospitalization period. In the univariate analysis, not receiving antibiotic therapy was associated with severe illness (OR:2.04; 95% IC: 1.109 to 3.77) (Table 3) and UCI admission (OR: 2.05; 95% IC: 1.03 to 4.07).

**Fig 1:**
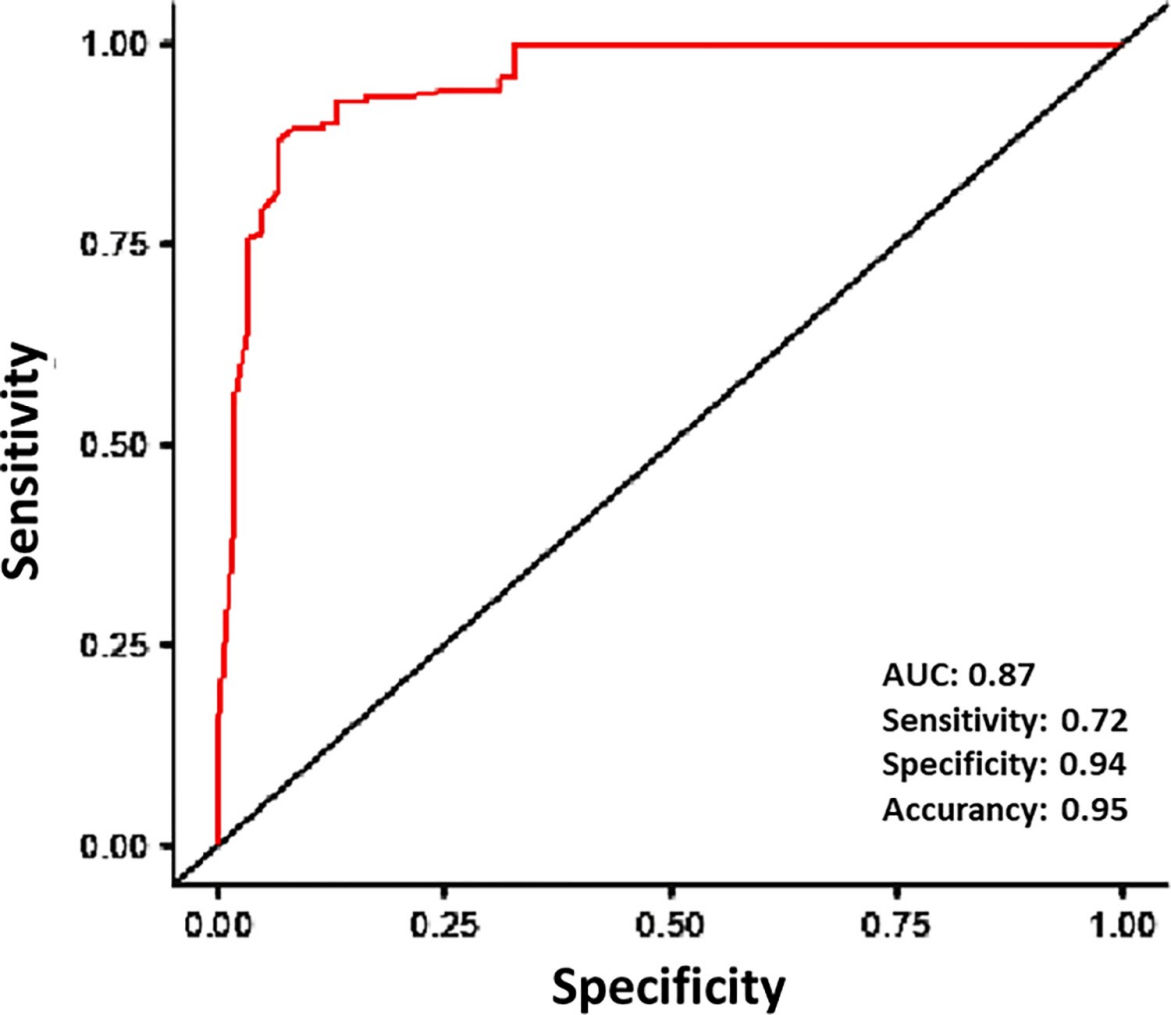
ROC curve for severe leptospirosis.

The multivariate analysis suggested dyspnea (OR 5.54; 95% CI: 1.46–20.98), tachycardia (OR 9.69; 95% CI: 15.96–58.88), and rash (OR 10.25; 95% CI: 25.01–42.08) were significantly associated with lab-confirmed severe leptospirosis (Table 3). The ROC curve analysis for the final logistic model showed good accuracy for severe leptospirosis cases as indicated by the AUC of 0.87 (Fig 1).

We found an overall mortality was 8.5% (n = 11), mostly among men (82.4%). The mean age for fatal cases was 47.8 (SD ±23.3) years. A large proportion of fatal cases reported having had contact with animals at home (70.6%) (Table 1). Fatal cases more frequently presented general symptoms as fever (99.5%), myalgias (87.5%), headache (83.7%), and complications such as jaundice (94.1%), thrombocytopenia (70.6%), and renal and liver failure (64.7%) (Table 2). The results from the univariate analysis showed a significant association between fatal cases and jaundice (OR: 13.15; 95% IC: 1.71 to 101.2), splenomegaly (OR: 4.65; 95% IC: 1.64–13.23), dyspnea (OR: 4.41; 95%IC: 1.55 to 12.54), renal failure (OR:5.05; 95% IC:1.77–14.39), liver failure (OR: 5.83; 95% IC: 2.04 to 16.68), and meningitis (OR: 8.04; 95% IC: 1.24 to 51.94). The results from the adjusted analysis show a statistically significant association between mortality and jaundice (OR: 11.96; 95% IC: 1.35 to 108.82).

## Discussion

This retrospective study evaluates the factors associated with the severity of leptospirosis among lab-confirmed human leptospirosis cases in Colombia between 2015 and 2020. We found that most cases occurred among men. Our results suggest that rash was the only symptom significantly associated with severe leptospirosis that would not typically require intensive management, as would other symptoms such as jaundice, dyspnea, or hemorrhage that were also significant.

In Colombia, few studies have been performed to describe the factor associated with severe leptospirosis, even though leptospirosis has been reported in several regions of the country [36,44]. This study was developed with data from passive surveillance of human leptospirosis, thus containing many biases. However, the results show a frequency of severe leptospirosis cases with around 30% of cases admitted to the ICU. Identifying the risk factors for severe leptospirosis could avoid patients’ complications and costs to the Colombian health system. Showing the information could help to raise awareness among local and regional physicians, so the leptospirosis infection can be quickly suspected and offer prompt treatment. Despite the limitations, these findings may be useful to focus future studies. Considering that recently human leptospirosis was the third in the list of prioritized zoonotic disease of greatest One Health concern in Colombia [45].

Most cases of leptospirosis reported in our data correspond to men, consistent with previous literature [1,10]. Bello et al., reported that men were 77% of human leptospirosis cases in Colombia [36]. The reasons for a higher prevalence and more severe manifestations of leptospirosis among men are not well understood. The higher prevalence among men has been explained because leptospirosis is an occupational hazard, associated with work activities outdoors and in contact with animals, such as farming, raising livestock, and the military, often associated with men in Colombia [46,47]. There is some evidence [48,49] that males are at higher risk than women for developing severe disease following leptospirosis infection. However, studies among athletes with similar environmental exposure have found no gender differences in the probability of developing illness [50,51].

Previous studies about the clinical factors associated with severe leptospirosis have shown signs and symptoms like hemorrhage [33], pulmonary signs and symptoms (abnormal lung sounds, dyspnea) [33], hypotension, oliguria, jaundice [52,53], and hyperkalemia [33]. Laboratory testing as packed cell volume (PCV: 29.8%), hyponatremia (<131 mEq/L), and highest alanine transaminase (ALT: 70 IU/L) were found to be independently associated with severe disease [52,54]. We found rash, dyspnea, tachycardia, and jaundice associated with severe leptospirosis.

Rash was significantly associated with disease severity. However, rash is not a common symptom in leptospirosis infections; it is often associated with other illnesses such as dengue, rickettsiosis, and chikungunya [55–57]. For example, a review estimated that 11–53% of dengue virus infections present rash compared to 5% of leptospirosis cases [58]. Studies in Puerto Rico [59], Thailand [52], and Nicaragua [38] did not find evidence that rash was an indicator of severe or fatal leptospirosis. In India, one study found that skin rash was significantly associated with leptospirosis in children [60], and another study found only suggestive evidence of such an association among urban leptospirosis cases [61], but not a sign of severity. However, previous evidence in Colombia suggested that rash is more common in other infections and may even be used, for example, to differentiate between dengue and leptospirosis [44]. Even though rash is not commonly reported in association with severe leptospirosis, our results showed that 22.4% of patients presented rash, of which 44.8% were severe. Because of the retrospective study design, we could not fully establish the characteristics of the rash in each case. We used data reported by the medical personnel who attended the case in each medical center and reported rash or exanthema in the patient.

Dyspnea was reported in 28.8% of the leptospirosis cases analyzed, with higher prevalence among severe (57.5%), ICU admissions (72.1%), and fatal (58.8%) cases. In human leptospirosis, lung involvement has been described in 20% to 70% of cases [62]. Pulmonary symptoms occur in both jaundiced patients and related symptoms, ranging from chest pain, cough, dyspnea, and hemoptysis, to acute respiratory distress syndrome [62,63]. These findings are important because the manifestations of pulmonary involvement in leptospirosis are often not recognized in endemic regions [62]. Other studies have also associated acute respiratory distress syndrome with severe leptospirosis and death [64]. However, in our study, we only used the information reported by the medical staff. We estimate the importance of adding the pulmonary radiographic alteration, but this information was impossible to obtain for this study.

We found that jaundice was associated with leptospirosis deaths. Previous studies have reported that jaundice is associated with about 19% of deaths in leptospirosis cases [9]. Liver injury is common in leptospirosis. Mild elevation of liver enzymes in the immune stage is usually evident, and serum bilirubin and alkaline phosphatase may be elevated. Jaundice is a symptom described as a severe complication of leptospirosis [38,65]. Jaundice and kidney failure have been associated with significantly higher mortality [9]. Nevertheless, jaundice is a frequent syndrome in infectious diseases, including malaria, toxoplasmosis, schistosomiasis, typhoid fever, Borrelia burgdorferi, scrub typhus, viral hepatitis, Ebola virus, hantavirus, and herpes. Understanding the local epidemiology of leptospirosis and other infections is necessary for formulating adequate differential diagnoses [66]. The inclusion of leptospirosis in the differential diagnosis represents one of the most critical challenges in Colombia because clinicians often focus on other more frequent febrile syndromes in the country [42,67–69]. Therefore, training and experience in clinical epidemiological reasoning may help health workers make safer decisions, including considering potential exposures to rodents and other mammals, surface water, extreme rainfall and flooding, and recreational or occupational activities associated with leptospirosis [66].

About one-third of reported lab-confirmed human leptospirosis cases in Colombia required admission to the ICU (30.3%) in 2015–2020. ICU admission in this study is reported in countries where leptospirosis is a health concern, such as China, were an estimated 40% of cases are admitted to the ICU [70]. Unfortunately, despite being an endemic country, human leptospirosis in Colombia is not widely known. We hope that our study will help raise awareness of the importance of leptospirosis in the country and that future prospective studies can track patient evolution and characterize epidemiological and behavioral risk factors. We could only analyze severe cases with ICU admission and deaths since these cases are discussed in analysis units in epidemiological surveillance.

Meningitis was described in a low proportion of analyzed cases (2.5%). Detecting meningitis requires a high level of clinical awareness because it presents various clinical signs and is usually aseptic [71–73]. Few studies report the proportion of meningitis caused by Leptospira spp. in endemic settings [71] or associated with leptospirosis mortality [9], potentially resulting in untimely diagnosis and targeted therapy of meningitis. This finding highlights the importance of considering leptospirosis as a differential diagnosis in patients with meningitis in Colombia and evaluating the history of cases with suspected meningitis, regarding occupational, recreational, or work history for possible exposure to *Leptospira* spp.

WHO treatment guidelines recommend administering antibiotics for leptospirosis regardless of the stage or severity of the disease [14]. For example, ceftriaxone has been reported as a protective factor for ICU admission [74]. Two-thirds (66.7%) of leptospirosis cases in our sample received antibiotic treatment. In the univariate analysis, non-antibiotic administration was associated with severe illness and UCI admission. Although delays in diagnosis may increase morbidity and mortality [66], not administering antibiotics may be linked to misrecognition of leptospirosis infection [42] because dengue and other infections are more common than leptospirosis in Colombia.

One of the major strengths of this study was the use of national data, including cases from all country with information on clinical outcomes (hospitalization, ICU admission, and death) and various clinical presentations. The study also has some limitations. First, our study was based on retrospective passive leptospirosis surveillance data. A better characterization of leptospirosis cases would result in more precise estimates. A prospective dataset could have included predictive factors before hospital admission, a more detailed description of clinical treatment (e.g., initiation of antibacterial therapy, days of hospitalization), variables that could support early detection (e.g., biochemical blood levels, such as bilirubin, leukocytes), and epidemiological and behavioral risk factors. Although the clinical features and laboratory results associated with the severe form of the disease are not yet well established, they have been linked to changes in blood chemistry [34,75], such as increased neutrophils [24]. Other laboratory tests, such as white cell count or creatinine, may have prognostic utility but were not available in our data. Electrocardiographic findings or chest X-rays were not available in the medical records and could have helped understand cases of leptospirosis associated with pneumonia. Second, passive surveillance depends on the patient presenting to healthcare, which results in several non-reported infections, particularly mild cases. Further, patients are reported at a single point in time, so patients reported as non-severe may have later developed a more severe illness that was not reported. Third, the study is limited to cases reported to national laboratory surveillance. These cases represent only a fraction of leptospirosis infections cases in Colombia, as suggested by previous studies in Colombia’s central and Caribbean regions [42,67,69]. Last, our analysis is subject to omitted variable bias as an observational study.

In conclusion, we used predictive models to identify factors associated with severe leptospirosis, admission to ICU, and mortality in laboratory-confirmed cases in Colombia. We hope our results can help physicians recognize clinical conditions associated with severe leptospirosis for more adequate and rapid patient triaging and timely treatment and management. These results should raise epidemiological and clinical awareness of human leptospirosis in Colombia. Leptospirosis remains a major clinical challenge, especially in tropical areas, and particularly for severe forms of the disease that can rapidly progress to multiple organ failure and death. Subsequent studies could prospectively analyze cases, including specific leptospirosis clinical scoring tools [19] to facilitate the recognition and triage of patients with a greater probability of deteriorating rapidly to reduce complications, health expenses, and prevent avoidable suffering and disease burden.

## Supporting information

S1 Table Serovars distribution in severe, ICU admission and non-survivors cases. (DOCX)

S1 File Epidemiological human Leptospirosis form. (PDF)
