# Supplementary material for: Demographic and clinical risk factors associated with severity of lab-confirmed human leptospirosis in Colombia, 2015–2020
Source: PLoS Negl Trop Dis. 2023 Jul 5;17(7):e0011454. doi: 10.1371/journal.pntd.0011454 (PMC10351742; doi:10.1371/journal.pntd.0011454)
Supplement: S1 Table — (DOCX) [file pntd.0011454.s001.docx]

**Supplementary Table S1. Serovars distribution in severe, ICU admission and non-survivors cases.**

| **Serovars** | **Total n (%)** | **Severe leptospirosis** | **Non-severe leptospirosis** | **ICU admission** | **Non-ICU admission** | **Survivors** | **Non-survivors** |
| --- | --- | --- | --- | --- | --- | --- | --- |
| Australis | 45 (22.4) | 18 (22.7) | 27 (23.7) | 13 (21.3) | 32 (22.9) | 4 (23.5) | 41 (22.3) |
| Ballum | 21 (10.4) | 11 (12.6) | 10 (8.8) | 9 (14.8) | 12 (8.6) | 1 (5.9) | 20 (10.9) |
| Autumnalis | 18 (9.0) | 10 (11.5) | 8 (7.0) | 6 (9.8) | 12 (8.6) | 2 (11.8) | 16 (8.7) |
| Wolfii | 13 (6.5) | 4 (4.6) | 9 (7.9) | 3 (4.9) | 10 (7.1) | 1 (5.9) | 12 (6.5) |
| Tarasovi | 12 (6.0) | 5 (5.7) | 7 (6.1)) | 3 (4.9) | 9 (6.4) | 0 (0.0) | 12 (6.5) |
| Icterohaemorrhagiae | 11 (5.5) | 6 (6.9) | 5 (4.4) | 5 (8.2) | 6 (4.3) | 2 (11.8) | 9 (4.9) |
| Pyrogenes | 9 (4.5) | 1 (1.1) | 8 (7.0) | 0 (0.0) | 9 (6.4) | 0 (0.0) | 9 (4.9) |
| Bataviae | 7 (3.5) | 2 (2.3) | 5 (4.4) | 1 (1.6) | 6 (4.3) | 1 (5.9) | 6 (3.3) |
| Hebdomadis | 6 (3.0) | 3 (3.4) | 3 (2.6) | 1 (1.6) | 5 (3.6) | 1 (5.9) | 5 (2.7) |
| Canicola | 5 (2.5) | 3 (3.4) | 2 (1.8) | 2 (3.3) | 3 (2.1) | 0 (0.0) | 5 (2.7) |
| Copenhageni | 5 (2.5) | 2 (2.3) | 3 (2.6) | 2 (3.3) | 3 (2.1) | 1 (5.9) | 4 (2.2) |
| Poi | 5 (2.5) | 4 (4.6) | 1 (0.9) | 4 (6.6) | 1 (0.7) | 1 (5.9) | 4 (2.2) |
| Proechimys | 5 (2.5) | 4 (4.6) | 1 (0.9) | 3 (4.9) | 2 (1.4) | 0 (0.0) | 5 (2.7) |
| Grippotyphosa | 4 (2.0) | 1 (1.1) | 3 (2.6) | 1 (1.6) | 3 (2.1) | 0 (0.0) | 4 (2.2) |
| Patoc | 4 (2.0) | 1 (1.1) | 3 (2.6) | 1 (1.6) | 3 (2.1) | 0 (0.0) | 4 (2.2) |
| Andamana | 3 (1.5) | 1 (1.1) | 2 (1.8) | 1 (1.6) | 2 (1.4) | 0 (0.0) | 3 (1.6) |
| Birkini | 3 (1.5) | 1 (1.1) | 2 (1.8) | 1 (1.6) | 2 (1.4) | 1 (5.9) | 2 (1.1) |
| Bratislava | 3 (1.5) | 1 (1.1) | 2 (1.8) | 1 (1.6) | 2 (1.4) | 0 (0.0) | 3 (1.6) |
| Castellonis | 3 (1.5) | 1 (1.1) | 2 (1.8) | 1 (1.6) | 2 (1.4) | 0 (0.0) | 3 (1.6) |
| Hardjo | 3 (1.5) | 0 (0.0) | 3 (2.6) | 0 (0.0) | 2 (1.4) | 0 (0.0) | 3 (1.6) |
| Javanica | 3 (1.5) | 2 (2.3) | 1 (0.9) | 1 (1.6) | 2 (1.4) | 1 (5.9) | 2 (1.1) |
| Panama | 3 (1.5) | 2 (2.3) | 1 (0.9) | 1 (1.6) | 2 (1.4) | 0 (0.0) | 3 (1.6) |
| Cynopteri | 2 (1.0) | 0 (0.0) | 2 (1.8) | 0 (0.0) | 2 (1.4) | 0 (0.0) | 2 (1.1) |
| Guaricura | 2 (1.0) | 1 (1.1) | 1 (0.9) | 0 (0.0) | 2 (1.4) | 0 (0.0) | 2 (1.1) |
| Pomona | 2 (1.0) | 0 (0.0) | 2 (1.8) | 0 (0.0) | 2 (1.4) | 0 (0.0) | 2 (1.1) |
| Rachmati | 2 (1.0) | 2 (2.3) | 0 (0.0) | 1 (1.6) | 1 (0.7) | 1 (5.9) | 1 (0.5) |
| Sejroe | 2 (1.0) | 1 (1.1) | 1 (0.9) | 0 (0.0) | 2 (1.4) | 0 (0.0) | 2 (1.1) |
