# Supplementary material for: Demographic and clinical risk factors associated with severity of lab-confirmed human leptospirosis in Colombia, 2015–2020
Source: PLoS Negl Trop Dis. 2023 Jul 5;17(7):e0011454. doi: 10.1371/journal.pntd.0011454 (PMC10351742; doi:10.1371/journal.pntd.0011454)
Supplement: S1 File — (PDF) [file pntd.0011454.s002.pdf]

Leptospirosis código INS: 455

La ficha de notificación es para fines de vigilancia en salud pública y todas las entidades que participen en el proceso deben garantizar la confidencialidad de la información LEY 1273/09 y 1266/09

Descripción del evento: la leptospirosis es una zoonosis de distribución mundial más frecuente en países tropicales, la cual inicia como un cuadro febril inespecífico, acompañado principalmente de cefalea y mialgias, solamente el 10% de los casos cursan con ictericia. Se puede presentar insuficiencia renal, hepática y hemorragia pulmonar aguda, complicaciones que son responsables de los casos de muerte. El diagnóstico presuntivo se establece con los síntomas más un antecedente epidemiológico de riesgo, la prueba de ELISA apoya el inicio del manejo médico, sin embargo la confirmación se realiza con micro aglutinación en muestras pareadas.

RELACIÓN CON DATOS BÁSICOS — **FOR-R02.0000-014 V:04 AÑO 2015**

A. Nombres y apellidos del paciente	B. Tipo de ID*	C. Número de identificación

*RC : REGISTRO CIVIL | TI : TARJETA IDENTIDAD | CC : CÉDULA CIUDADANÍA | CE : CÉDULA EXTRANJERÍA |- PA : PASAPORTE | MS : MENOR SIN ID | AS : ADULTO SIN ID

5. DATOS CLÍNICOS

5.1. Signos y síntomas (marque con X los que se presenten)
- ☐ Fiebre
- ☐ Mialgias
- ☐ Cefalea
- ☐ Hepatomegalia
- ☐ Ictericia

6. ANTECEDENTES EPIDEMIOLÓGICOS

6.1 ¿Hay animales en la casa? (Marque con una X los que tenga)
- ☐ 1. Perros
- ☐ 2. Gatos
- ☐ 3. Bovinos
- ☐ 4. Equinos
- ☐ 5. Porcinos
- ☐ 6. Ninguno
- ☐ 7. Otros

6.1.1 ¿Cuál otro? ____________________

6.2 ¿Contacto con animales enfermos en los últimos 6 meses?
- ○ 1. Sí
- ○ 2. No

6.3 ¿Ha visto ratas dentro o alrededor de su domicilio?
- ○ 1. Sí
- ○ 2. No

6.4 ¿Ha visto ratas dentro o alrededor de su lugar de trabajo?
- ○ 1. Sí
- ○ 2. No

6.5 Fuentes de agua
- ☐ 1. Acueducto
- ☐ 2. Pozo comunitario
- ☐ 3. Río
- ☐ 4. Tanque de almacenamiento

6.6 ¿Alcantarillas destapadas cerca del domicilio o sitio de trabajo?
- ○ 1. Sí
- ○ 2. No

6.7 ¿Inundaciones en la zona en los últimos 30 días?
- ○ 1. Sí
- ○ 2. No

6.8 ¿Contacto con aguas estancadas durante los últimos 30 días
- ○ 1. Sí
- ○ 2. No

6.9 Antecedentes de actividades deportivas, de baño o pesca en los últimos 30 días antes del comienzo de los síntomas en:
- ☐ 1. Represa
- ☐ 2. Río
- ☐ 3. Arroyo
- ☐ 4. lago/laguna
- ☐ 5. Sin antecedente

6.10 Disposición de residuos sólidos
- ○ 1. Recolección
- ○ 2. Disposición peridomiciliaria

6.11 Tiempo de almacenamiento de la basura en casa
- ○ Entre 1 a 3 días
- ○ Entre 4 a 7 días
- ○ 3. Más de 7 días

6.12 ¿Conoce personas con sintomatología similar en la misma vivienda durante los últimos 30 días
- ○ 1. Sí
- ○ 2. No

Correos: sivigila@ins.gov.co / ins.sivigila@gmail.com

INSTRUCTIVO DILIGENCIAMIENTO FICHAS DE NOTIFICACIÓN DATOS COMPLEMENTARIOS
LEPTOSPIROSIS (Cód INS: 455)

VARIABLE	CATEGORÍAS Y DEFINICIÓN	CRITERIOS SISTEMATIZACIÓN	OBLIG
**A. Nombres y apellidos del paciente<br>B. Tipo de ID<br>C. Número de identificación**	* Se relaciona con el tipo de documento de identidad que tiene el paciente que está siendo notificados. Diligencie una sola opción en esta variable.	* El tipo de documento debe ser coincidente con la edad del paciente.<br>* El tipo y número de documento debe coincidir con la información ingresada en la ficha de datos básicos.	**SI**
**5. DATOS CLÍNICOS**			
**5.1 Signos y síntomas**	Variable con múltiples opciones de respuesta. Corresponde a los principales signos y síntomas que están presentes y permiten sospechar leptospirosis	Diligencie la variable, de lo contrario el sistema no permitirá cotinuar con el ingreso de la información.	**SI**
**6. ANTECEDENTES EPIDEMIOLÓGICOS**			
**6.1 ¿Hay animales en la casa?**	Variable con múltiples opciones de respuesta. Marque con una X los animales que habitan en la casa o finca donde vive el paciente	Para la sistematización de la variable, debe reconocer si el caso convive o no con animales, lo cual deberá reflejarse con los criterios de 1 = Si ó 2 = No.	**SI**
**6.1.1 ¿Cuál otro?**	Diligencie el espacio siempre y cuando en la variable 6.1 haya marcado la opción 7 = Otro.	Depende de la respuesta en la variable 6.1, opción 7 = Otros.	**NO**
**6.2 ¿Contacto con animales enfermos en los últimos 6 meses?**	Marque con una X la opción según corresponda.<br>1 = Si<br>2 = No	Diligencie la variable, de lo contrario el sistema no permitirá cotinuar con el ingreso de la información.	**SI**
**6.3 ¿Ha visto ratas dentro o alrededor de su domicilio?**	Marque con una X la opción según corresponda.<br>1 = Si<br>2 = No	Diligencie la variable, de lo contrario el sistema no permitirá cotinuar con el ingreso de la información.	**SI**
**6.4 ¿Ha visto ratas dentro o alrededor de su lugar de trabajo?**	Marque con una X la opción según corresponda.<br>1 = Si<br>2 = No	Diligencie la variable, de lo contrario el sistema no permitirá cotinuar con el ingreso de la información.	**SI**
**6.5 Fuentes de agua**	Variable con múltiples opciones de respuesta. Marque con una X teniendo en cuenta las diferentes fuentes de agua con las que preparan alimentos o para baño.	Para la sistematización de la variable, debe reconocer las fuentes de agua que refiere el caso, lo cual deberá reflejarse con los criterio 1 = Si ó 2 = No.	**SI**
**6.6 ¿Alcantarillas destapadas cerca del domicilio o sitio de trabajo?**	Marque con una X la opción según corresponda.<br>1 = Si<br>2 = No	Diligencie la variable, de lo contrario el sistema no permitirá cotinuar con el ingreso de la información.	**SI**
**6.7 ¿Inundaciones en la zona en los últimos 30 días?**	Marque con una X la opción según corresponda.<br>1 = Si<br>2 = No<br>Tenga en cuenta que las inundaciones se presenten en el sitio de vivienda del paciente	Diligencie la variable, de lo contrario el sistema no permitirá cotinuar con el ingreso de la información.	**SI**
**6.8 ¿Contacto con aguas estancadas durante los últimos 30 días?**	Marque con una X la opción según corresponda.<br>1 = Si<br>2 = No<br>Incluye piscinas, pozos, tanques, lagunas, lodos	Diligencie la variable, de lo contrario el sistema no permitirá cotinuar con el ingreso de la información.	**SI**
**6.9 Antecedentes de actividades deportivas, de baño o pesca en los últimos 30 días antes del comienzo de los síntomas en:**	Variable con múltiples opciones de respuesta. Marque con una X las opciones según correspondan.	Para la sistematización de la variable, debe reconocer si el caso realizó alguna actividad deportiva acuática en el periodo mencionado, lo cual deberá verse reflejado con las opciones 1 = Si ó 2 = No.	**SI**
**6.10 Disposición de residuos sólidos**	Marque con una X la opción según corresponda.<br>1 = Recolección<br>2 = Disposición domiciliaria	Diligencie la variable, de lo contrario el sistema no permitirá cotinuar con el ingreso de la información.	**SI**
**6.11 Tiempo de almacenamiento de basura en casa**	Marque con una X la opción según corresponda.<br>1 = 1-3 días<br>2 = 4-7 días<br>3 = Más de 7 días	Diligencie la variable, de lo contrario el sistema no permitirá cotinuar con el ingreso de la información.	**SI**
**6.12 Conoce personas con sintomatología similar en la misma vivienda durante los últimos 30 días**	Marque con una X la opción según corresponda.<br>1 = Si<br>2 = No	Diligencie la variable, de lo contrario el sistema no permitirá cotinuar con el ingreso de la información.	**SI**
